# 2-(3,5-Dioxo-4-aza­tri­cyclo­[5.2.1.0^2,6^]dec-8-en-4-yl)acetic acid

**DOI:** 10.1107/S1600536813021764

**Published:** 2013-08-10

**Authors:** Mehmet Akkurt, Aliasghar Jarrahpour, Pouria Shirvani, Muhammad Nawaz Tahir

**Affiliations:** a Department of Physics, Faculty of Sciences, Erciyes University, 38039 Kayseri, Turkey; b Department of Chemistry, College of Sciences, Shiraz University, 71454 Shiraz, Iran; c Department of Physics, University of Sargodha, Sargodha, Pakistan

## Abstract

The asymmetric unit of the title compound, C_11_H_11_NO_4_, contains two mol­ecules, *A* and *B*, with different conformations: in mol­ecule *A*, the norborne and carb­oxy­lic acid groups lie to the same side of the heterocycle, whereas in a mol­ecule *B*, they lie on opposite sides. In the crystal, the *A* mol­ecules form *R*_2_^2^(8) carb­oxy­lic acid inversion dimers, linked by pairs of O—H⋯O hydrogen bonds. The *B* mol­ecules link to one of the ketone O atoms of the *A* mol­ecule by an O—H⋯O inter­action, resulting in tetra­mers (two *A* and two *B* mol­ecules). The tetra­mers are linked by weak C—H⋯O inter­actions, generating a three-dimensional network.

## Related literature

For a related structure, see: Bartkowska *et al.* (1997 ▶). For further synthetic details, see: Biagini *et al.* (1995 ▶).
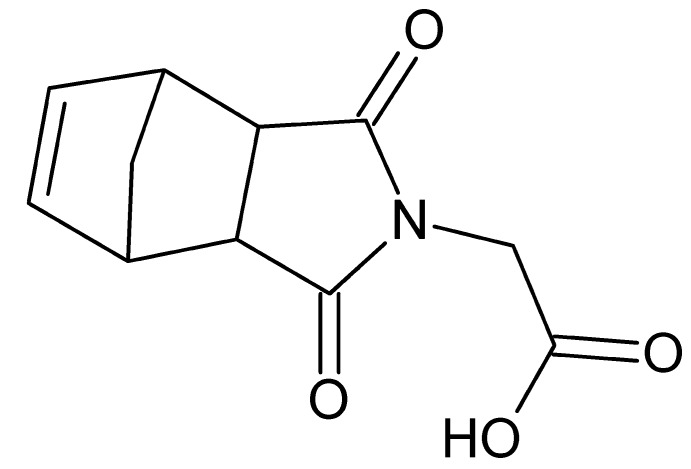


## Experimental

### 

#### Crystal data

C_11_H_11_NO_4_
*M* *_r_* = 221.21
Triclinic, 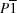

*a* = 6.5060 (3) Å
*b* = 11.8417 (4) Å
*c* = 14.1794 (5) Å
α = 104.385 (2)°
β = 97.905 (2)°
γ = 99.549 (2)°
*V* = 1025.07 (7) Å^3^
*Z* = 4
Mo *K*α radiation
μ = 0.11 mm^−1^
*T* = 296 K
0.28 × 0.20 × 0.16 mm

#### Data collection

Bruker Kappa APEXII CCD diffractometer
Absorption correction: multi-scan (*SADABS*; Bruker, 2005 ▶) *T*_min_ = 0.970, *T*_max_ = 0.983
15543 measured reflections
3985 independent reflections
3245 reflections with *I* > 2σ(*I*)
*R*_int_ = 0.032

#### Refinement

*R*[*F* ^2^ > 2σ(*F* ^2^)] = 0.040
*wR*(*F* ^2^) = 0.107
*S* = 1.03
3985 reflections
292 parameters
H-atom parameters constrained
Δρ_max_ = 0.30 e Å^−3^
Δρ_min_ = −0.21 e Å^−3^

### 

Data collection: *APEX2* (Bruker, 2009 ▶); cell refinement: *SAINT* (Bruker, 2009 ▶); data reduction: *SAINT*; program(s) used to solve structure: *SHELXS97* (Sheldrick, 2008 ▶); program(s) used to refine structure: *SHELXL97* (Sheldrick, 2008 ▶); molecular graphics: *ORTEP-3 for Windows* (Farrugia, 2012 ▶) and *PLATON* (Spek, 2009 ▶); software used to prepare material for publication: *WinGX* (Farrugia, 2012 ▶) and *PLATON* (Spek, 2009 ▶).

## Supplementary Material

Crystal structure: contains datablock(s) global, I. DOI: 10.1107/S1600536813021764/hb7113sup1.cif

Structure factors: contains datablock(s) I. DOI: 10.1107/S1600536813021764/hb7113Isup2.hkl

Supplementary material file. DOI: 10.1107/S1600536813021764/hb7113Isup3.cml

Additional supplementary materials:  crystallographic information; 3D view; checkCIF report

## Figures and Tables

**Table 1:** Hydrogen-bond geometry (Å, °)

*D*—H⋯*A*	*D*—H	H⋯*A*	*D*⋯*A*	*D*—H⋯*A*
O1—H1⋯O2^i^	0.82	1.84	2.6504 (18)	170
O5—H5*A*⋯O3^ii^	0.82	1.86	2.6509 (18)	163
C11—H11⋯O8^iii^	0.93	2.57	3.440 (2)	156
C15—H15⋯O8^iv^	0.98	2.33	3.201 (2)	147
C16—H16⋯O1^iv^	0.98	2.48	3.1473 (19)	125

Symmetry codes: (i) 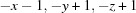; (ii) 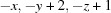; (iii) 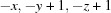; (iv) 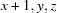.

**Fig. 1.**
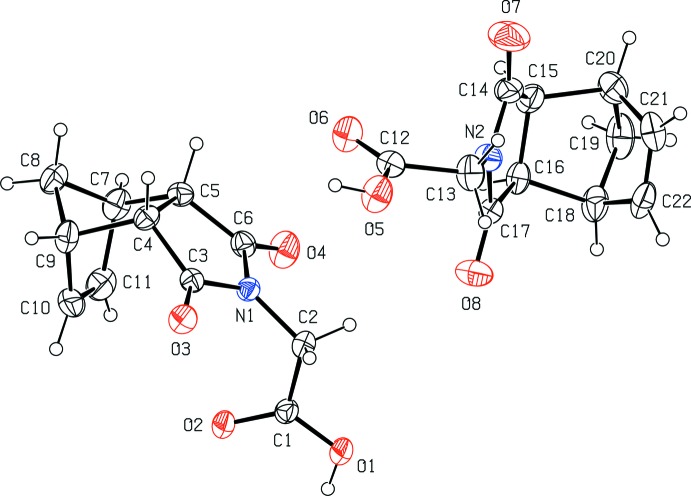
View of the two molecules of the title compound in the asymmetric unit. Displacement ellipsoids for non-H atoms are drawn at the 30% probability level.

**Fig. 2.**
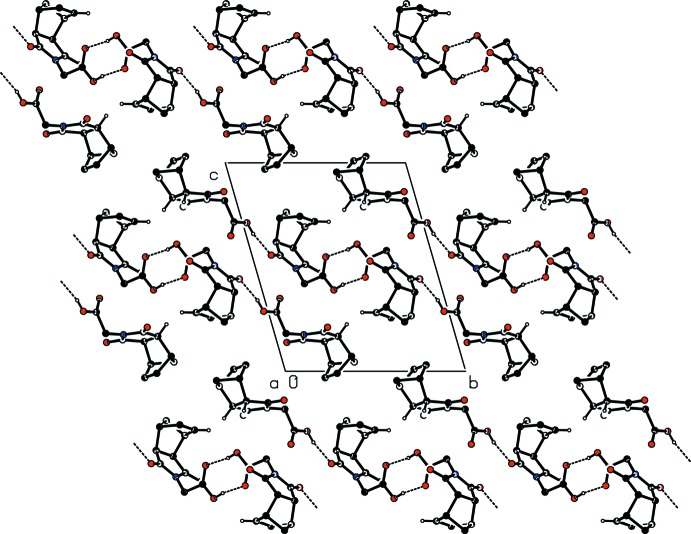
View of the dimer and C—H···O hydrogen bonds of the title compound along the a axis.

## supplementary crystallographic information

### 1. Comment

As shown in Fig. 1, the asymmetric unit of the title compound contains two independently molecules 1 (with N1) and 2 (with N2). The norbornene units of the molecules 1 and 2 are bound *endo* with respect to acetic acid. The sum of the three C—N—C angles at the imide N atom is 359.61 (13)° for molecule 1 and 359.88 (15) ° for molecule 2. In molecule 1, the N1—C2 bond length [1.448 (2) Å] is longer than the N1—C3 [1.3663 (19) Å] and N1—C6 [1.4044 (19) Å] bond lengths. In molecule 2, the corresponding bond lengths are N2—C13 of 1.442 (2) Å, N2—C17 of 1.377 (2) Å and N2—C14 of 1.384 (2) Å, respectively. As expected, this indicates a delocalized π-electron system along the imide parts of the molecules, as in a similar structure (Bartkowska *et al.*, 1997).

In the crystal, pairs of molecules generate a dimer of the *R*^2^_2_(8) motif by O—H···O hydrogen bonds; these two molecules are linked to the other two molecules by O—H···O hydrogen bonds (Table 1, Fig. 2). In addition, C—H···O hydrogen bonds contribute to the overall crystal packing.

### 2. Experimental

To *endo*-5-norbornene-2,3-dicarboxylic anhydride (16.41 g, 100.0 mmol) dissolved in DMF (30 ml) was added glycine (7.50 g, 100.0 mmol). The reaction mixture was refluxed for 24 h, coolded to room temperature, diluted with ethyl acetate (70 ml), and washed with saturated aqueous ammonium chloride solution (5×50 ml). The organic phase was dried on anhydrous Na_2_SO_4_, filtered and evaporated *in vacuo*. The residue was recrystallized (5 times) from etheyl acetate giving *N*-5-norbornene-2,3-dicarboxyloylglycine as a white crystalline solid (yield 61%); mp: 422–424 K (Biagini *et al.*, 1995).

### 3. Refinement

All H atoms were geometrically placed [(O—H = 0.82 Å (hydroxyl), C—H = 0.93 Å (aromatic), C—H = 0.97 Å (methylene) and C—H = 0.98 Å (methine)] and refined as riding with *U*_iso_(H) = 1.5*U*_eq_(O) for the hydroxyl group and 1.2*U*_eq_(C) for the others.

### Crystal data

**Table:**

C_11_H_11_NO_4_	*Z* = 4
*M**_r_* = 221.21	*F*(000) = 464
Triclinic, *P*1	*D*_x_ = 1.433 Mg m^−^^3^
Hall symbol: -P 1	Mo *K*α radiation, λ = 0.71073 Å
*a* = 6.5060 (3) Å	Cell parameters from 318 reflections
*b* = 11.8417 (4) Å	θ = 3.1–22.5°
*c* = 14.1794 (5) Å	µ = 0.11 mm^−^^1^
α = 104.385 (2)°	*T* = 296 K
β = 97.905 (2)°	Plate, colourless
γ = 99.549 (2)°	0.28 × 0.20 × 0.16 mm
*V* = 1025.07 (7) Å^3^	

### Data collection

**Table:**

Bruker Kappa APEXII CCD diffractometer	3985 independent reflections
Radiation source: fine-focus sealed tube	3245 reflections with *I* > 2σ(*I*)
Graphite monochromator	*R*_int_ = 0.032
ω scans	θ_max_ = 26.0°, θ_min_ = 1.5°
Absorption correction: multi-scan (*SADABS*; Bruker, 2005)	*h* = −8→8
*T*_min_ = 0.970, *T*_max_ = 0.983	*k* = −14→12
15543 measured reflections	*l* = −17→17

### Refinement

**Table:**

Refinement on *F*^2^	Secondary atom site location: difference Fourier map
Least-squares matrix: full	Hydrogen site location: inferred from neighbouring sites
*R*[*F*^2^ > 2σ(*F*^2^)] = 0.040	H-atom parameters constrained
*wR*(*F*^2^) = 0.107	*w* = 1/[σ^2^(*F*_o_^2^) + (0.0458*P*)^2^ + 0.3164*P*] where *P* = (*F*_o_^2^ + 2*F*_c_^2^)/3
*S* = 1.03	(Δ/σ)_max_ < 0.001
3985 reflections	Δρ_max_ = 0.30 e Å^−^^3^
292 parameters	Δρ_min_ = −0.21 e Å^−^^3^
0 restraints	Extinction correction: *SHELXL97* (Sheldrick, 2008), FC^*^=KFC[1+0.001XFC^2^Λ^3^/SIN(2Θ)]^-1/4^
Primary atom site location: structure-invariant direct methods	Extinction coefficient: 0.031 (3)

### Special details

**Table:**

Geometry. Bond distances, angles *etc*. have been calculated using the rounded fractional coordinates. All su's are estimated from the variances of the (full) variance-covariance matrix. The cell e.s.d.'s are taken into account in the estimation of distances, angles and torsion angles
Refinement. Refinement on *F*^2^ for ALL reflections except those flagged by the user for potential systematic errors. Weighted *R*-factors *wR* and all goodnesses of fit *S* are based on *F*^2^, conventional *R*-factors *R* are based on *F*, with *F* set to zero for negative *F*^2^. The observed criterion of *F*^2^ > σ(*F*^2^) is used only for calculating -*R*-factor-obs *etc*. and is not relevant to the choice of reflections for refinement. *R*-factors based on *F*^2^ are statistically about twice as large as those based on *F*, and *R*-factors based on ALL data will be even larger.

### Fractional atomic coordinates and isotropic or equivalent isotropic displacement parameters (Å^2^)

**Table:**

	*x*	*y*	*z*	*U*_iso_*/*U*_eq_	
O1	−0.35166 (19)	0.60085 (12)	0.60965 (8)	0.0532 (4)	
O2	−0.34179 (18)	0.59322 (10)	0.45144 (8)	0.0457 (4)	
O3	−0.18607 (17)	0.89261 (10)	0.44282 (8)	0.0444 (4)	
O4	0.2190 (2)	0.64574 (12)	0.52862 (10)	0.0615 (5)	
N1	0.00738 (18)	0.77624 (10)	0.50666 (9)	0.0330 (3)	
C1	−0.2816 (2)	0.64006 (14)	0.54167 (11)	0.0370 (5)	
C2	−0.1179 (3)	0.75396 (14)	0.57948 (11)	0.0398 (5)	
C3	−0.0349 (2)	0.84335 (12)	0.44353 (10)	0.0326 (4)	
C4	0.1310 (2)	0.84417 (13)	0.37965 (11)	0.0367 (4)	
C5	0.2676 (2)	0.75925 (14)	0.40793 (11)	0.0392 (5)	
C6	0.1738 (2)	0.71746 (14)	0.48685 (11)	0.0386 (5)	
C7	0.2420 (3)	0.66135 (15)	0.30761 (13)	0.0483 (6)	
C8	0.2424 (3)	0.74010 (16)	0.23690 (13)	0.0518 (6)	
C9	0.0474 (3)	0.78668 (16)	0.26604 (12)	0.0488 (6)	
C10	−0.0999 (3)	0.67172 (19)	0.25673 (13)	0.0596 (7)	
C11	0.0136 (3)	0.59804 (16)	0.28153 (14)	0.0595 (6)	
O5	0.2685 (2)	1.02427 (13)	0.71240 (10)	0.0613 (5)	
O6	0.4902 (2)	0.91401 (12)	0.65030 (10)	0.0590 (5)	
O7	0.8866 (3)	0.94587 (15)	0.86157 (14)	0.0927 (7)	
O8	0.2460 (2)	0.69605 (13)	0.78880 (11)	0.0682 (5)	
N2	0.5459 (2)	0.83974 (12)	0.82437 (10)	0.0422 (4)	
C12	0.4055 (3)	0.95414 (13)	0.71640 (12)	0.0413 (5)	
C13	0.4395 (3)	0.93605 (16)	0.81844 (13)	0.0537 (6)	
C14	0.7635 (3)	0.85293 (17)	0.84901 (14)	0.0524 (6)	
C15	0.8085 (3)	0.73603 (18)	0.85732 (13)	0.0520 (6)	
C16	0.5937 (3)	0.65205 (14)	0.83248 (11)	0.0439 (5)	
C17	0.4369 (3)	0.72531 (14)	0.81157 (11)	0.0399 (5)	
C18	0.5844 (3)	0.60936 (17)	0.92804 (13)	0.0589 (7)	
C19	0.8155 (4)	0.60299 (19)	0.95558 (15)	0.0725 (9)	
C20	0.8975 (3)	0.73413 (19)	0.96526 (15)	0.0611 (7)	
C21	0.7602 (4)	0.79025 (19)	1.03067 (13)	0.0604 (7)	
C22	0.5757 (4)	0.7165 (2)	1.00967 (13)	0.0620 (7)	
H1	−0.44970	0.54320	0.58450	0.0800*	
H2A	−0.18820	0.81980	0.59930	0.0480*	
H2B	−0.02410	0.75070	0.63770	0.0480*	
H4	0.21650	0.92460	0.39260	0.0440*	
H5	0.41620	0.80040	0.43240	0.0470*	
H7	0.34670	0.61050	0.30410	0.0580*	
H8A	0.21900	0.69480	0.16780	0.0620*	
H8B	0.36930	0.80250	0.25300	0.0620*	
H9	−0.00640	0.83860	0.22880	0.0590*	
H10	−0.24660	0.65520	0.23680	0.0720*	
H11	−0.03840	0.52050	0.28270	0.0710*	
H5A	0.26000	1.04120	0.65950	0.0920*	
H13A	0.52280	1.00910	0.86500	0.0650*	
H13B	0.30300	0.91990	0.83800	0.0650*	
H15	0.90050	0.70730	0.81150	0.0620*	
H16	0.58550	0.58470	0.77470	0.0530*	
H18	0.47950	0.53660	0.92080	0.0710*	
H19A	0.86390	0.55010	0.90330	0.0870*	
H19B	0.84650	0.58260	1.01740	0.0870*	
H20	1.05070	0.76360	0.98820	0.0730*	
H21	0.79850	0.86410	1.07780	0.0720*	
H22	0.45980	0.72830	1.03990	0.0740*	

### Atomic displacement parameters (Å^2^)

**Table:**

	*U*^11^	*U*^22^	*U*^33^	*U*^12^	*U*^13^	*U*^23^
O1	0.0539 (8)	0.0664 (8)	0.0356 (6)	−0.0085 (6)	0.0056 (5)	0.0229 (6)
O2	0.0508 (7)	0.0526 (7)	0.0333 (6)	0.0019 (5)	0.0073 (5)	0.0175 (5)
O3	0.0453 (7)	0.0463 (6)	0.0503 (7)	0.0215 (5)	0.0128 (5)	0.0195 (5)
O4	0.0726 (9)	0.0697 (9)	0.0627 (8)	0.0401 (7)	0.0168 (7)	0.0368 (7)
N1	0.0329 (6)	0.0351 (6)	0.0331 (6)	0.0077 (5)	0.0074 (5)	0.0124 (5)
C1	0.0351 (8)	0.0458 (8)	0.0362 (8)	0.0120 (6)	0.0090 (6)	0.0189 (7)
C2	0.0453 (9)	0.0434 (8)	0.0334 (8)	0.0094 (7)	0.0113 (7)	0.0131 (6)
C3	0.0350 (8)	0.0269 (7)	0.0339 (7)	0.0048 (6)	0.0035 (6)	0.0077 (6)
C4	0.0413 (8)	0.0308 (7)	0.0405 (8)	0.0061 (6)	0.0126 (7)	0.0125 (6)
C5	0.0313 (8)	0.0454 (9)	0.0413 (8)	0.0097 (6)	0.0073 (6)	0.0112 (7)
C6	0.0368 (8)	0.0416 (8)	0.0383 (8)	0.0130 (7)	0.0032 (6)	0.0117 (7)
C7	0.0581 (11)	0.0466 (9)	0.0472 (9)	0.0235 (8)	0.0203 (8)	0.0116 (7)
C8	0.0610 (11)	0.0535 (10)	0.0441 (9)	0.0114 (8)	0.0228 (8)	0.0128 (8)
C9	0.0600 (11)	0.0591 (10)	0.0361 (9)	0.0240 (9)	0.0126 (8)	0.0199 (8)
C10	0.0478 (11)	0.0783 (14)	0.0369 (9)	−0.0007 (10)	0.0035 (8)	−0.0012 (9)
C11	0.0791 (14)	0.0413 (9)	0.0468 (10)	−0.0051 (9)	0.0195 (9)	−0.0011 (8)
O5	0.0718 (9)	0.0707 (9)	0.0633 (8)	0.0422 (7)	0.0204 (7)	0.0363 (7)
O6	0.0687 (9)	0.0656 (8)	0.0545 (8)	0.0322 (7)	0.0190 (7)	0.0223 (6)
O7	0.0668 (10)	0.0869 (11)	0.1180 (14)	−0.0239 (9)	−0.0078 (9)	0.0567 (10)
O8	0.0395 (8)	0.0742 (9)	0.0841 (10)	0.0003 (6)	0.0055 (7)	0.0205 (8)
N2	0.0412 (8)	0.0410 (7)	0.0485 (8)	0.0115 (6)	0.0040 (6)	0.0203 (6)
C12	0.0409 (9)	0.0343 (8)	0.0495 (9)	0.0085 (7)	0.0041 (7)	0.0151 (7)
C13	0.0706 (12)	0.0478 (10)	0.0494 (10)	0.0263 (9)	0.0084 (9)	0.0178 (8)
C14	0.0411 (10)	0.0630 (11)	0.0569 (11)	0.0010 (8)	0.0048 (8)	0.0324 (9)
C15	0.0426 (10)	0.0765 (12)	0.0533 (10)	0.0278 (9)	0.0183 (8)	0.0327 (9)
C16	0.0615 (11)	0.0407 (8)	0.0338 (8)	0.0192 (8)	0.0130 (7)	0.0106 (7)
C17	0.0405 (9)	0.0451 (9)	0.0351 (8)	0.0077 (7)	0.0103 (7)	0.0119 (7)
C18	0.0848 (14)	0.0483 (10)	0.0469 (10)	0.0084 (9)	0.0091 (9)	0.0244 (8)
C19	0.1114 (19)	0.0709 (14)	0.0522 (11)	0.0530 (13)	0.0125 (11)	0.0271 (10)
C20	0.0496 (11)	0.0810 (14)	0.0597 (12)	0.0251 (10)	−0.0006 (9)	0.0307 (10)
C21	0.0796 (15)	0.0617 (12)	0.0375 (9)	0.0257 (11)	−0.0025 (9)	0.0094 (8)
C22	0.0779 (14)	0.0836 (14)	0.0408 (10)	0.0317 (12)	0.0274 (10)	0.0281 (10)

### Geometric parameters (Å, º)

**Table:**

O1—C1	1.275 (2)	C4—H4	0.9800
O2—C1	1.2391 (18)	C5—H5	0.9800
O3—C3	1.2240 (18)	C7—H7	0.9800
O4—C6	1.201 (2)	C8—H8A	0.9700
O1—H1	0.8200	C8—H8B	0.9700
O5—C12	1.320 (2)	C9—H9	0.9800
O6—C12	1.190 (2)	C10—H10	0.9300
O7—C14	1.206 (3)	C11—H11	0.9300
O8—C17	1.208 (2)	C12—C13	1.507 (2)
O5—H5A	0.8200	C14—C15	1.491 (3)
N1—C6	1.4044 (19)	C15—C16	1.515 (3)
N1—C3	1.3663 (19)	C15—C20	1.567 (3)
N1—C2	1.448 (2)	C16—C18	1.565 (2)
N2—C14	1.384 (2)	C16—C17	1.490 (3)
N2—C17	1.377 (2)	C18—C19	1.520 (3)
N2—C13	1.442 (2)	C18—C22	1.505 (3)
C1—C2	1.499 (2)	C19—C20	1.521 (3)
C3—C4	1.5018 (19)	C20—C21	1.489 (3)
C4—C9	1.564 (2)	C21—C22	1.310 (4)
C4—C5	1.537 (2)	C13—H13A	0.9700
C5—C7	1.564 (2)	C13—H13B	0.9700
C5—C6	1.494 (2)	C15—H15	0.9800
C7—C11	1.500 (3)	C16—H16	0.9800
C7—C8	1.530 (3)	C18—H18	0.9800
C8—C9	1.532 (3)	C19—H19A	0.9700
C9—C10	1.494 (3)	C19—H19B	0.9700
C10—C11	1.313 (3)	C20—H20	0.9800
C2—H2B	0.9700	C21—H21	0.9300
C2—H2A	0.9700	C22—H22	0.9300
			
C1—O1—H1	109.00	C9—C10—H10	126.00
C12—O5—H5A	109.00	C11—C10—H10	126.00
C2—N1—C6	121.45 (13)	C10—C11—H11	126.00
C2—N1—C3	125.13 (13)	C7—C11—H11	126.00
C3—N1—C6	113.03 (12)	O5—C12—C13	108.83 (15)
C14—N2—C17	113.13 (15)	O6—C12—C13	126.08 (17)
C13—N2—C17	122.36 (15)	O5—C12—O6	125.09 (16)
C13—N2—C14	124.39 (15)	N2—C13—C12	113.30 (15)
O1—C1—O2	125.28 (15)	O7—C14—C15	128.9 (2)
O1—C1—C2	114.07 (13)	N2—C14—C15	107.95 (16)
O2—C1—C2	120.63 (14)	O7—C14—N2	123.17 (19)
N1—C2—C1	112.87 (12)	C14—C15—C20	114.56 (16)
O3—C3—N1	122.95 (13)	C16—C15—C20	103.08 (15)
O3—C3—C4	128.30 (13)	C14—C15—C16	105.36 (16)
N1—C3—C4	108.75 (12)	C15—C16—C18	102.94 (14)
C5—C4—C9	103.06 (13)	C17—C16—C18	115.33 (15)
C3—C4—C5	104.78 (12)	C15—C16—C17	105.32 (15)
C3—C4—C9	115.48 (12)	O8—C17—N2	122.86 (17)
C6—C5—C7	115.17 (14)	O8—C17—C16	128.93 (17)
C4—C5—C7	102.83 (12)	N2—C17—C16	108.20 (15)
C4—C5—C6	105.18 (11)	C16—C18—C19	99.66 (16)
O4—C6—N1	122.08 (14)	C16—C18—C22	106.71 (16)
N1—C6—C5	107.86 (13)	C19—C18—C22	99.66 (17)
O4—C6—C5	130.05 (14)	C18—C19—C20	93.79 (17)
C5—C7—C8	99.37 (14)	C15—C20—C19	99.08 (16)
C5—C7—C11	106.49 (15)	C15—C20—C21	107.11 (17)
C8—C7—C11	100.24 (15)	C19—C20—C21	100.74 (18)
C7—C8—C9	93.90 (14)	C20—C21—C22	107.46 (19)
C8—C9—C10	100.08 (15)	C18—C22—C21	107.9 (2)
C4—C9—C8	99.71 (13)	N2—C13—H13A	109.00
C4—C9—C10	105.91 (14)	N2—C13—H13B	109.00
C9—C10—C11	108.17 (17)	C12—C13—H13A	109.00
C7—C11—C10	107.79 (17)	C12—C13—H13B	109.00
N1—C2—H2A	109.00	H13A—C13—H13B	108.00
C1—C2—H2A	109.00	C14—C15—H15	111.00
C1—C2—H2B	109.00	C16—C15—H15	111.00
N1—C2—H2B	109.00	C20—C15—H15	111.00
H2A—C2—H2B	108.00	C15—C16—H16	111.00
C9—C4—H4	111.00	C17—C16—H16	111.00
C3—C4—H4	111.00	C18—C16—H16	111.00
C5—C4—H4	111.00	C16—C18—H18	116.00
C6—C5—H5	111.00	C19—C18—H18	116.00
C7—C5—H5	111.00	C22—C18—H18	116.00
C4—C5—H5	111.00	C18—C19—H19A	113.00
C11—C7—H7	116.00	C18—C19—H19B	113.00
C8—C7—H7	116.00	C20—C19—H19A	113.00
C5—C7—H7	116.00	C20—C19—H19B	113.00
C7—C8—H8B	113.00	H19A—C19—H19B	110.00
H8A—C8—H8B	110.00	C15—C20—H20	116.00
C9—C8—H8B	113.00	C19—C20—H20	116.00
C7—C8—H8A	113.00	C21—C20—H20	116.00
C9—C8—H8A	113.00	C20—C21—H21	126.00
C10—C9—H9	116.00	C22—C21—H21	126.00
C8—C9—H9	116.00	C18—C22—H22	126.00
C4—C9—H9	116.00	C21—C22—H22	126.00
			
C3—N1—C6—C5	6.13 (17)	C4—C5—C7—C8	38.05 (16)
C2—N1—C6—C5	179.33 (13)	C8—C7—C11—C10	−32.68 (19)
C3—N1—C2—C1	94.00 (18)	C5—C7—C11—C10	70.38 (19)
C6—N1—C2—C1	−78.35 (18)	C5—C7—C8—C9	−59.54 (15)
C2—N1—C3—O3	0.3 (2)	C11—C7—C8—C9	49.25 (16)
C6—N1—C3—O3	173.21 (14)	C7—C8—C9—C4	58.76 (15)
C2—N1—C3—C4	−179.72 (13)	C7—C8—C9—C10	−49.47 (15)
C6—N1—C3—C4	−6.82 (16)	C4—C9—C10—C11	−69.91 (19)
C3—N1—C6—O4	−172.69 (15)	C8—C9—C10—C11	33.32 (18)
C2—N1—C6—O4	0.5 (2)	C9—C10—C11—C7	−0.4 (2)
C14—N2—C17—C16	1.95 (18)	O5—C12—C13—N2	166.23 (15)
C13—N2—C14—C15	174.18 (15)	O6—C12—C13—N2	−15.0 (3)
C14—N2—C13—C12	91.2 (2)	O7—C14—C15—C16	−179.5 (2)
C17—N2—C14—C15	−2.1 (2)	N2—C14—C15—C16	1.29 (19)
C14—N2—C17—O8	−179.16 (16)	N2—C14—C15—C20	−111.26 (18)
C13—N2—C17—C16	−174.38 (14)	O7—C14—C15—C20	68.0 (3)
C13—N2—C17—O8	4.5 (2)	C14—C15—C16—C17	−0.18 (17)
C17—N2—C14—O7	178.64 (19)	C14—C15—C20—C21	47.5 (2)
C17—N2—C13—C12	−92.86 (19)	C16—C15—C20—C19	37.85 (19)
C13—N2—C14—O7	−5.1 (3)	C16—C15—C20—C21	−66.4 (2)
O1—C1—C2—N1	161.11 (14)	C20—C15—C16—C18	−0.96 (19)
O2—C1—C2—N1	−20.5 (2)	C14—C15—C16—C18	−121.38 (15)
N1—C3—C4—C9	117.19 (14)	C20—C15—C16—C17	120.24 (15)
O3—C3—C4—C9	−62.8 (2)	C14—C15—C20—C19	151.75 (18)
N1—C3—C4—C5	4.57 (15)	C15—C16—C17—O8	−179.80 (17)
O3—C3—C4—C5	−175.46 (15)	C15—C16—C18—C22	66.9 (2)
C5—C4—C9—C10	67.62 (16)	C17—C16—C18—C19	−150.46 (16)
C3—C4—C5—C6	−0.97 (15)	C17—C16—C18—C22	−47.2 (2)
C5—C4—C9—C8	−35.88 (16)	C15—C16—C17—N2	−1.00 (17)
C3—C4—C9—C8	−149.51 (14)	C18—C16—C17—O8	−67.1 (2)
C3—C4—C9—C10	−46.00 (19)	C18—C16—C17—N2	111.74 (16)
C3—C4—C5—C7	119.94 (13)	C15—C16—C18—C19	−36.34 (18)
C9—C4—C5—C6	−122.17 (13)	C16—C18—C19—C20	59.13 (16)
C9—C4—C5—C7	−1.26 (15)	C22—C18—C19—C20	−49.82 (17)
C4—C5—C6—N1	−2.81 (16)	C16—C18—C22—C21	−69.3 (2)
C4—C5—C6—O4	175.88 (17)	C19—C18—C22—C21	33.9 (2)
C6—C5—C7—C11	48.16 (18)	C18—C19—C20—C15	−59.50 (16)
C6—C5—C7—C8	151.85 (14)	C18—C19—C20—C21	49.99 (17)
C7—C5—C6—O4	63.5 (2)	C15—C20—C21—C22	70.5 (2)
C4—C5—C7—C11	−65.65 (16)	C19—C20—C21—C22	−32.6 (2)
C7—C5—C6—N1	−115.25 (15)	C20—C21—C22—C18	−0.8 (2)

### Hydrogen-bond geometry (Å, º)

**Table:**

*D*—H···*A*	*D*—H	H···*A*	*D*···*A*	*D*—H···*A*
O1—H1···O2^i^	0.82	1.84	2.6504 (18)	170
O5—H5*A*···O3^ii^	0.82	1.86	2.6509 (18)	163
C11—H11···O8^iii^	0.93	2.57	3.440 (2)	156
C15—H15···O8^iv^	0.98	2.33	3.201 (2)	147
C16—H16···O1^iv^	0.98	2.48	3.1473 (19)	125

Symmetry codes: (i) −*x*−1, −*y*+1, −*z*+1; (ii) −*x*, −*y*+2, −*z*+1; (iii) −*x*, −*y*+1, −*z*+1; (iv) *x*+1, *y*, *z*.
